# Factors related to resilience in nursing workers during and after the pandemic: a prospective cohort

**DOI:** 10.1590/0034-7167-2025-0396

**Published:** 2026-09-21

**Authors:** Luciana Olino, Miguel Lucas Silva da Paixão, Patrícia Bitencourt Toscani Greco, Tânia Solange Bosi de Souza Magnago, Daiane Dal Pai, Juliana Petri Tavares

**Affiliations:** I Universidade Federal do Rio Grande do Sul. Porto Alegre, Rio Grande do Sul, Brazil; II Universidade Federal do Rio Grande. Rio Grande, Rio Grande do Sul, Brazil; III Universidade Federal de Santa Maria. Santa Maria, Rio Grande do Sul, Brazil

**Keywords:** Resilience, Nursing, COVID-19, Cohort Studies, Occupational Health., Resiliencia, Enfermería, COVID-19, Estudios de Cohortes, Salud Laboral.

## Abstract

**Objective:**

to analyze the factors that influence resilience at work in nursing professionals who worked in hospital settings during and after the COVID-19 pandemic.

**Methods:**

a prospective multicenter cohort study with 163 nursing workers during and after the pandemic. Sociodemographic, work-related, health, and resilience variables were collected using the Resilience at Work Scale Brazil-25. Bivariate analysis and linear regression modeling were performed.

**Results:**

mean resilience scores were similar in 2020 (73.3±11.1) and 2022 (72.9±11.2). Regarding the score, each additional year of age adds 0.27 points to the Resilience at Work Scale; being female decreases 10.06 points; having a mental illness decreases 4.67 points; rating sleep as very poor decreases 6.1 points; holding a supervisory position decreases 13.53 points; working in sectors specifically for COVID-19 victims increases 6.90 points.

**Conclusion:**

although similar in both periods, different factors influenced the resilience scores at work.

## RESUMEN

**Objetivo:**

analizar los factores que influyen en la resiliencia laboral en profesionales de enfermería que trabajaron en el ámbito hospitalario durante y después de la pandemia de COVID-19.

**Métodos:**

estudio de cohorte multicéntrico prospectivo con 163 profesionales de enfermería durante y después de la pandemia. Se recopilaron variables sociodemográficas, laborales, de salud y de resiliencia mediante la Resilience at Work Scale Brasil-25. Se realizó un análisis bivariado y un modelo de regresión lineal.

**Resultados:**

las puntuaciones medias de resiliencia fueron similares en 2020 (73,3±11,1) y 2022 (72,9 ±11,2). En cuanto a la puntuación, cada año adicional de edad añade 0,27 puntos a la Escala de Resiliencia Laboral; ser mujer disminuye 10,06 puntos; padecer una enfermedad mental disminuye 4,67 puntos; calificar el sueño como muy deficiente disminuye 6,1 puntos; ocupar un puesto de liderazgo disminuye 13,53 puntos; y trabajar en sectores específicos para víctimas de la COVID-19 aumenta 6,90 puntos.

**Conclusión:**

aunque similares en ambos períodos, diferentes factores influyeron en las puntuaciones de resiliencia laboral.

## INTRODUCTION

The daily routine of nursing staff is marked by a high workload, resulting from frequent understaffing, overcrowded services, and the need to establish therapeutic bonds that guarantee comprehensive and safe patient care. This context directly impacts workers’ physical and mental health, increasing their vulnerability to the development of musculoskeletal and psychological disorders^(1)^.

During the COVID-19 pandemic, the healthcare system collapsed due to the increased number and complexity of cases. This situation resulted in an overload of work for healthcare professionals, especially nursing staff, who make up the majority of human resources in healthcare services. This scenario led to fatigue, stress, and psychological distress among these professionals^(2-5)^.

In this context, nurses and technicians needed to find adaptation strategies to continue practicing their profession. In this regard, resilience stands out, understood as the capacity to overcome adversity and unpleasant events, as well as the ability to successfully adapt to changes and uncertainties. Classic models of resilience at work emphasize individual factors, such as coping and emotional self-regulation, while contemporary approaches broaden the concept to include organizational and contextual dimensions, such as institutional support, working conditions, professional autonomy, and the quality of interpersonal relationships. Furthermore, resilience contributes to reducing emotional exhaustion, improving commitment, and enhancing the ability to cope with the adversities inherent in the profession^(2,3,6-9)^.

Beyond the pandemic context, nursing staff already faced challenges in developing resilience at work. Despite the challenging scenarios faced by these workers, cohort studies assessing their resilience at work during and after the pandemic are still scarce in the literature. A Brazilian study^(10)^ highlights that, before the pandemic, 15.2% of the nursing staff presented low levels of resilience. However, there is no consensus in the literature that allows us to determine how these levels behaved in the post-pandemic period, highlighting an important gap in scientific knowledge.

Given this context, it becomes necessary to deepen our understanding of the importance of resilience in the workplace, which has been characterized by work overload and impacts on physical and mental health. Furthermore, although the impacts of the COVID-19 pandemic on workers’ mental health are widely documented, gaps remain regarding the factors associated with resilience and its evolution throughout the post-pandemic period, justifying the present study.

## OBJECTIVES

To analyze the factors that influenced resilience at work in nursing workers who worked in hospital settings during and after the COVID-19 pandemic.

## METHODS

### Ethical aspects

This research was approved by the Research Ethics Committee and complies with Resolution 466/12 of the Brazilian National Health Council, with Resolution 510/16 on the use of data obtained directly from participants, and with General Data Protection Law guidelines, as participants’ privacy was maintained. The Informed Consent Form was made available to participants via email before they filled out the forms.

### Study design, period, and location

This is a multicenter, analytical, quantitative cohort study guided by the STrengthening the Reporting of OBservational studies in Epidemiology tool^(11)^. Participants were invited to participate in the study during the pandemic peak (phase 1), when there was an increase in demand for hospital care for patients with the disease, from August to October 2020. In this stage, 817 professionals participated. In the second phase (phase 2), which took place from August to December 2022, with the stabilization of the pandemic curve characterized by a decrease in new cases, hospitalizations, and deaths from the virus, in addition to population immunization that makes the pandemic controlled, 382 nursing workers completed the questionnaire.

The study included four hospital institutions in the state of Rio Grande do Sul, Brazil, which had their workflows adapted to care for patients affected by COVID-19. These institutions include two general and teaching hospitals in Porto Alegre (HA, 784 beds; HC, 850 beds), a public hospital specializing in acute trauma also located in Porto Alegre (HB, 237 beds), and a general and teaching hospital located in Santa Maria (HD, 403 beds).

### Population, sample, inclusion and exclusion criteria

The study population consisted of 6,899 nursing workers. Nursing workers (nurses, technicians, and nursing assistants) working in hospital care during the pandemic who responded to the questionnaire in both data collection periods (during and after the pandemic) were included in the study. Those professionals who were absent for more than 30 days during the data collection period were excluded.

A sample size of 150 participants was calculated for each year in order to test whether there is a difference in work resilience levels between the pandemic peak phase (2020) and the post-pandemic period (2022). The calculation considered a power of 80%, a significance level of 5%, and percentages of 32.2% (pre-pandemic) and 48.5% (during the pandemic), respectively, as reported in the literature^(12,13)^. The cohort study sample consisted of 163 nursing staff members, i.e., individuals who participated in both phases of the study.

### Study protocol

The data collection instrument was created using Google Forms^®^, addressing sociodemographic variables such as sex, age (date of birth), race or ethnicity, number of children, and marital status. Other variables relate to lifestyle and health habits, such as smoking, increased alcohol consumption, physical activity, sleep quality, medication use, having any illness, and self-assessment of the impact of the pandemic on physical and mental health. The form was sent to professionals via email, with the employee’s email contact accessed through institutional authorization. Additionally, there was internal dissemination of information within the departments of the participating hospitals.

Work-related data was collected, including length of time in the profession, job title, type of employment contract, work at another institution, whether the employee holds a supervisory position, work shift, department, whether they work in the specific unit for COVID-19 victims, whether they were reassigned during the pandemic, whether they cared for a COVID-19 patient, whether the work demands increased, the level of fear felt regarding exposure to the risk of contamination, and whether the employee needed to take time off work for health reasons and due to suspected or confirmed COVID-19.

The Resilience at Work Scale (RAW Scale) Brazil-25, validated in Brazil^(7)^, was used to assess resilience at work. It is a seven-point Likert scale, with options from 0 (Strongly disagree) to 6 (Strongly agree). It assesses resilience at work through the means of responses across seven domains: living authentically; finding your calling; maintaining perspective; managing stress; interacting cooperatively; staying healthy; and building networks^(6)^. The scale’s internal consistency and its domains was assessed using Cronbach’s alpha coefficient, showing a reliability of 0.83. Composite reliability ranged from 0.56 to 0.85 across domains^(7)^. The cut-off points defined by the author of RAW Scale Brasil-25 were used, whose permission for use was granted after contacting her. Since this information is protected by copyright, it cannot be reproduced in its entirety in this document.

### Static analyses

Data from both collections were captured from Google Forms^®^ and sent for analysis using the Statistical Package for the Social Sciences^®^ version 18. The Shapiro-Wilk test was used to test for normality. Categorical variables were presented as absolute and relative frequencies, and continuous variables as central tendency and dispersion. Student’s t-test was used to compare symmetrical continuous variables between two independent groups, and the paired t-test was used for dependent groups. Parametric variables with three or more groups were subjected to analysis of variance.

In multiple analysis, linear regression was used, with robust variance expressed as a prevalence ratio, and its respective 95% Confidence Intervals (95%CI). The criterion for including variables in the final multiple regression model was having a p-value <0.20 for both the exposure (associated factors) and the outcome (resilience at work). Data with a 95%CI or a two-tailed p-value less than 0.05 were considered statistically significant differences.

## RESULTS

The study included 163 nursing workers, predominantly female (n=139; 85%) and white (n=130; 79.8%) in both phases. The median age was 43 (39-49) years in 2020 and 44 (39-48) years in 2022. In 2020, 117 (71.8%) professionals had children, increasing to 120 (73.6%) in 2022. Before the pandemic, 126 (77.3%) were married, and after the pandemic, this number decreased to 124 (76.1%).

In relation to work variables, 57.7% (n=94) were nurses, and 42.3% (n=69) were nursing technicians and assistants. As for the sector, the majority worked in adult inpatient units, with 46 (28.2%) in 2020 and 47 (28.8%) in 2022. The smallest percentage of workers (n=56; 34.4%) were not reassigned from their sector during the pandemic in 2020, and 16 (9.81%) in 2022. In 2020, the majority worked at the HC institution (n=85; 52.2%) on the day shift (n=120; 73.6%), under a contract regime according to the Consolidation of Labor Laws (n=139; 85.3%) and without activity in another institution (n=147; 90.2%). In 2022, work at the HC institution also prevailed (n=86; 52.8%) during the day shift (n=109; 66.9%), under a contract in accordance with the Consolidation of Labor Laws (n=138; 84.7%) and without activity in another institution (n=150; 92%).

As for resilience at work, in 2020, the mean for workers was 73.3 ±11.1; of these, 125 (76.7%) showed high resilience at work, and 38 (23.3%) showed low resilience at work. In 2022, the mean value was 72.9 (±11.2). Of these, 122 (74.8%) workers demonstrated high resilience at work, and 41 (25.2%) demonstrated low resilience. Table 1 presents the distribution of resilience at work (RAW Scale Brazil-25) of nursing workers (n=163) working during and after the pandemic, according to sociodemographic characteristics, lifestyle habits, and health.

**Table 1:** Distribution of mean resilience scores (RAW Scale Brazil-25) of nursing staff (N=163) working during and after the pandemic according to sociodemographic characteristics, lifestyle habits, and health, Porto Alegre, Rio Grande do Sul, Brazil, 2020 and 2022

Sociodemographic variables, lifestyle habits, and health		Year	
		2020	2022
Age (years)^*^		R=0.19	R=0.20
		p=**0.017**	p=**0.011**
Sex	Female	72.33 ±11.29	71.88 ±12.23
	Male	78.28 ±9.28	73.19 ±11.96
		**p=0.008**	p=0.624
Color/race	White	73.21 ±10.63	72.29 ±10.69
	Not white	73.19 ±13.36	71.21 ±16.97
		p=0.993	p=0.650
Presence of children	Yes	73.17 ±10.58	72.06 ±12.43
	No	73.30 ±12.73	72.11 ±11.55
		p=0.949	p=0.982
Marital status	Single	73.28 ±10.44	72.60 ±12.23
	Married	73.19 ±11.44	71.91 ±12.19
		p=0.963	p=0.760
Smoking	Yes	77.27 ±8.25	73.23 ±9.39
	No	72.94 ±11.33	71.97 ±12.40
		p=0.145	p=0.659
Increased alcohol consumption in the last six months	Yes	73.18 ±10.58	69.61 ±13.17
	No	73.95 ±11.36	72.61 ±11.92
		p=0.149	p=0.266
Physical activity practiced in the last six months	Yes	77.96 ±8.30	73.55 ±12.14
	No	71.33 ±11.65	70.2 ±12.03
		p=**<0.001**	p=0.081
Medication started within the last six months	Yes	71.33 ±12.20	67.17 ±14.16
	No	73.9 ±10.77	73.67 ±11.04
		p=0.223	p=**0.003**
Presence of some disease	Yes	72.23 ±12.14	71.42 ±12.02
	No	74.31 ±9.99	72.86 ±12.37
		p=0.229	p=0.452
Cardiovascular diseases	Yes	74.41 ±11.17	73.56 ±11.91
	No	72.95 ±11.22	71.67 ±12.25
		p=0.525	p=0.411
Endocrine diseases	Yes	70.11 ±12.43	67.18 ±16.92
	No	73.45±11.10	72.5 ±11.64
		p=0.384	p=0.287
Mental illnesses	Yes	65.68 ±11.11	64.92 ±11.77
	No	74.44 ±10.75	73.62 ±11.73
		p=**0.001**	p=**0.001**
Musculoskeletal disorders	Yes	71.59 ±12.24	68.41 ±11.76
	No	73.54 ±10.98	73.04 ±12.13
		p=0.441	p=**0.048**
Digestive diseases	Yes	70.77 ±12.05	68.49 ±11.6
	No	73.54 ±11.07	72.55 ±11.62
		p=0.339	p=0.287
Respiratory diseases	Yes	73.21 ±11.45	70.86 ±9.83
	No	73.21 ±11.20	72.21 ±12.43
		p=0.998	p=0.608
Sleep quality over the last six months	Excellent	75.10 ±10.41	73.7 ±11.30
	Terrible	68.22 ±11.74	65.51 ±13.18
		p=**0.001**	p=**0.001**
Impact of the COVID-19 pandemic on physical health	Intense	72.04 ±11.11	70.63 ±12.51
	None	74.58 ±11.21	73.24 ±11.82
		p=0.150	p=0.176
Impact of the COVID-19 pandemic on mental health	Intense	71.33 ±11.22	70.34 ±11.91
	None	78.60 ±9.3	74.26 ±12.22
		p=**<0.001**	p=**0.042**

* *Pearson’s correlation values (R); p - p value.*

In 2020, the following were associated with lower mean resilience (p<0.05): younger age (R=0.19); female sex (72.33±11.29) compared to male sex (78.28±9.28); sedentary lifestyle (71.33±11.65); presence of mental illness (65.68±11.11); very poor sleep quality (68.22±11.74); and perception of intense impact of the pandemic on mental health (71.33±11.22).

In 2022, lower resilience scores were observed among younger workers (R=0.20), those who started medication in the last six months (67.17±14.16), those with mental (64.92±11.77) or musculoskeletal (68.41±11.76) illnesses, those with sleep rated as very poor (65.51±13.18), and those who reported a significant impact of the pandemic on their mental health (70.34±11.9). Workers’ work characteristics and resilience indices are detailed in Table 2.

**Table 2:** Distribution of mean work resilience scores (RAW Scale Brazil-25) of nursing workers (N=163) working during and after the pandemic according to work characteristics, Porto Alegre, Rio Grande do Sul, Brazil, 2020 and 2022

Job characteristics		Year	
		2020	2022
Years working in the profession^*^		R=0.07 p=0.392	R=0.05 p=0.496
Position	Nurse	72.57 ±11.29	72.02 ±10.91
	Nursing technician	73.77 ±10.38	72.3 ±13.73
	Nursing assistant	77.22 ±18.03	70.56 ±15.52
		p=0.612	p=0.944
Type of employment relationship	Private sector employee	73.59 ±11.13	72.32 ±11.81
	Statutory employee	70.64 ±11.69	70.72 ±14.15
		p=0.449	p=0.547
Works at another institution	Yes	73.92 ±12.77	73.84 ±12.21
	No	73.13 ±11.05	71.92 ±12.19
		p=0.815	p=0.586
Management position	Yes	77.16 ±9.45	72.70 ±9.6
	No	72.81 ±11.31	72 ±12.47
		p=0.113	p=0.778
Work shift	Day shift	72.96 ±11.83	71.42 ±12.26
	Night shift	75.21 ±8.7	74.41 ±12.26
	No fixed shift	68.96 ±10.18	67.5 ±8.56
		p=0.296	p=0.210
Work sector	Emergency Room	76.37 ±8.71	70.63 ±10.4
	Intensive Care Unit	72.21 ±12.74	74.43 ±9.53
	Inpatient Unit	72.01 ±9.95	73.79 ±12.9
	Surgical Center	77 ±8.86	73.46 ±10.31
	Pediatrics and Neonatology	70.17 ±14.09	69.28 ±12.39
	Other Departments	73.20 ±12.20	69.99 ±13.7
		p=0.444	p=0.532
Relocated from their sector within the last six months	Yes	73.64 ±11.54	65.79 ±12.41
	No	72.97 ±11.05	72.76 ±11.98
		p=0.724	p=**0.046**
Specific work sector for COVID-19 victims	Yes	72.43 ±10.66	77.60 ±11.84
	No	73.48 ±11.41	71.51 ±12.10
		p=0.591	p=0.075
Treated a patient with suspected or confirmed COVID-19 in the last six months	Yes	73.14 ±11.01	71.64 ±12.46
	No	73.68 ±11.82	74.07 ±10.70
		p=0.862	p=0.288
Fear felt in the face of exposure to the risk of contamination in the last six months	Intense	71.88 ±11.19	71.75 ±11.83
	Little	75.06 ±11.01	72.17 ±12.31
		p=0.075	p=0.851
Increased demands on the job over the past six months	Intense	73.23 ±10.41	72.08 ±11.34
	Little	72.9 ±13.96	72.06 ±13.57
		p=0.881	p=0.989
Absence from work due to health reasons in the last six months	Yes	71.79 ±11.93	68.93 ±12.54
	No	74.33 ±10.51	75.03 ±11.09
		p=0.157	p=**0.001**
Absence from work due to suspected COVID-19 in the last six months	Yes	73.29 ±11.53	68.56 ±11.71
	No	73.14 ±10.98	74.12 ±12.01
		p=0.934	p=**0.004**
Absence from work due to a COVID-19 diagnosis in the last six months	Yes	73.4 ±12.41	70.26 ±11.05
	No	73.18 ±11.05	72.79 ±12.55
		p=0.940	p=0.210

* *Pearson’s correlation values (R); p - p value.*

Having been reassigned from their sector in 2022, being absent from work due to health reasons, and suspected COVID-19 were associated with lower mean resilience scores (p<0.05). Table 3 presents the categorized levels of resilience at work.

**Table 3:** Nursing workers (N=163) active during and after the pandemic, according to high and low levels of resilience at work across the scale domains, Porto Alegre, Rio Grande do Sul, Brazil, 2020 and 2022

Domain		Year	
		2020	2022
Living authentically	High	95.1 (155)	93.3 (152)
	Low	4.9 (8)	6.7 (11)
Finding your calling	High	86.5 (141)	85.3 (139)
	Low	13.5 (22)	14.7 (24)
Maintaining perspective	High	20.9 (34)	25.2 (41)
	Low	79.1 (129)	74.8 (122)
Managing stress	High	60.7 (99)	60.1 (98)
	Low	39.3 (64)	39.9 (65)
Interacting cooperatively	High	73.6 (120)	76.1 (124)
	Low	26.4 (43)	23.9 (39)
Staying healthy	High	65.6 (107)	63.2 (103)
	Low	34.4 (56)	36.8 (60)
Building networks	High	80.4 (131)	76.7 (125)
	Low	19.6 (32)	23.3 (38)

Regarding resilience, in 2020, the mean score for workers was 72.1 (±12.2). In 2022, the mean value was 72.9 (±11.2). Table 3 presents the nursing workers active during and after the pandemic according to the resilience domains. The linear regression model for the selected resilience variables is presented in Table 4.

**Table 4:** Linear regression model for variables related to resilience at work (N=163), Porto Alegre, Rio Grande do Sul, Brazil 2022

Variables		2020	2022
		Prevalence ratio (95%CI)	Prevalence ratio (95%CI)
Age		0.14 (-0.25 a 0.53)	0.267 (0.06 a 0.48)
		p=0.471	p=**0.013**
Sex	Female	-10.06 (-18.85 a 1.27)	N/A
	Male	1	N/A
		p=**0.025**	N/A
Smoking	Yes	5.02 (-5.02 a 15.42)	N/A
	No	1	N/A
		p=0.319	N/A
Increased alcohol consumption in the last six months	Yes	3.83 (-1.51 a 9.17)	N/A
	No	1	N/A
		p=0.159	N/A
Physical activity practiced in the last six months	Yes	1	1
	No	-7.35 (-0.35 - 15.1)	-1.87 (-1.53 a -5.27)
		p=0.062	p=0.282
Medication started within the last six months	Yes	N/A	-2.66 (-6.62 a 1.3)
	No	N/A	1
		N/A	p=0.188
Mental illnesses	Yes	-2.56 (-9 a 3.86)	-4.67 (-9.33 a -0.23)
	No	1	1
		p=0.435	p=**0.049**
Musculoskeletal disorders	Yes	N/A	-1.62 (-5.8 a 2.56)
	No	N/A	1
		N/A	0.447
Sleep quality over the last six months	Terrible	-1.82 (-7.51 a 3.86)	-6.08 (-10.88 a -1.27)
	Excellent	1	1
		p=0.530	p=**0.013**
The impact of the COVID-19 pandemic on the physical health	Intense	1.61 (-3.97 a 7.2)	0.8 (-2.89 a 4.49)
	None	1	1
		p=0.571	p=0.671
The impact of the COVID-19 pandemic on the mental health	Intense	2.19 (-8.9 - 13.29)	-1.45 (-5.21 a 2.31)
	None	1	1
		p=0.700	0.451
Management position	Yes	-13.53 (-26.08 a -0.99)	N/A
	No	1	N/A
		p=0.034	N/A
Relocated from his sector in the last six months	Yes	N/A	-3.62 (-9.2 a 1.95)
	No	N/A	1
		N/A	p=0.203
Specific work sector for COVID-19 victims	Yes	N/A	6.90 (1.22 a 12.58)
	No	N/A	1
		N/A	p=0.017
Fear felt in the face of exposure to the risk of contamination in the last six months	Intense	-3.75 (-10.69 a 3.20)	N/A
	Little	1	N/A
		p=0.290	N/A
Absence from work due to health reasons in the last six months	Yes	1.10 (-4.37 a 6.56)	-3.08 (-6.98 a 0.82)
	No	1	1
		p=0.694	p=0.122
Absence from work due to suspected COVID-19 in the last six months	Yes	Not applicable	-1.32 (-5.36 a 2.73)
	No	Not applicable	1
		Not applicable	p=0.523

*CI - Confidence Interval; p - p value; N/A - Not applicable.*

In the linear regression analysis of variables related to resilience, in 2020, being female decreased, on average, 10.06 points on the Resilience at Work Scale compared to being male (p=0.025). Holding a management position scored 13.53 points lower on the Resilience at Work Scale compared to those who did not hold a management position (p=0.034). In 2022, for each year of age increase, the individual obtained approximately 0.27 more points on the Resilience at Work Scale (p=0.013); having a mental illness decreased 4.67 points on the Resilience at Work Scale compared to those who did not have one (p=0.049); those who rated their sleep quality as very poor experienced a decrease of approximately 6.1 points on the Resilience at Work Scale compared to those who reported excellent sleep quality (p=0.013); working in sectors specifically for COVID-19 victims resulted in an mean of 6.90 points more on the Resilience at Work Scale compared to those who did not work (p=0.017).

## DISCUSSION

The global mean of resilience at work was slightly higher during the pandemic than after it ended. This likely occurred because workers needed to vehemently seek strength and resources to offset the damage they were experiencing in 2020, whereas by 2022 there was already stability and, consequently, less need for adaptation. A study conducted in Belgium, outside the pandemic context, detected lower resilience at work, as well as a lack of activities to reduce stress, which was directly associated with suicide attempts^(14)^. A study conducted in the pandemic context with nursing professionals from all regions of Brazil demonstrated that participants generally had higher mean scores for depression linked to low levels of resilience and self-efficacy^(15)^.

The prevalence of high and low levels of resilience at work remained similar across the scale’s domains during the pandemic and post-pandemic periods. The dimension with the highest score in both years was “Living authentically”, related to self-awareness, emotional regulation, and alignment of personal and institutional values, which favors mood and pressure management^(6)^. The domain with the lowest score was “Maintaining perspective”, which involves dealing with adversity and maintaining positivity in the face of negative situations-an aspect possibly hampered by the difficulties faced during and after the pandemic. Organizational support and planning are fundamental to strengthening this dimension^(6)^.

It became evident that, after the pandemic, mean resilience scores at work were higher among older individuals. These data are similar to a study conducted in Santa Catarina, Brazil, during the pandemic, which demonstrated a statistically significant difference in resilience scores among people over 35 years old compared to those in younger age groups^(16)^. It is possible to think that the greater the age and professional experience, the better the analytical capacity to overcome adversity and, consequently, the greater the repertoire of strategies for dealing with adversity, due to previously lived experiences.

In 2020, the worst levels of resilience at work were observed among women. A study on resilience described similar results, in which male professionals had statistically higher resilience scores than female workers. The nurse category had a statistically higher resilience score than the nursing technician category^(15)^.

Nursing professionals who did not engage in physical activity during the pandemic, as well as those who developed musculoskeletal disorders after the pandemic, showed lower mean levels of resilience at work. A study on resilience, conducted with the nursing staff of a general hospital, found that 26.6% of participants had low resilience; 40% had moderate resilience; 33.4% had high resilience; and only 33.3% of respondents regularly engaged in physical activity. The research also showed that the intensity of musculoskeletal pain ranged from moderate to high among participants, in different anatomical regions^(17)^.

Regarding the start of medication use, those professionals who began using medication after the pandemic showed lower mean resilience scores at work. A Brazilian study on resilience described that participants who had never used medication were the most resilient, with a statistically significant difference in the comparisons of scores^(18)^.

The benefit of resilience at work as a protective factor against psychiatric illnesses is well-established in the literature^(19)^. In the present study, the lowest mean resilience scores were found among workers who had mental illnesses, and having a mental illness in 2022 decreased points on the Resilience at Work Scale. The data support the study that showed a statistically significant negative correlation between depression and resilience at work, showing that the greater the resilience, the lower the depression^(20)^. The importance of developing resilient strategies as a tool for treating and preventing psychological illness was reinforced.

In addition to mental health issues, decreased resilience at work can affect sleep. In this study, professionals who showed lower mean resilience scores at work were those who rated their sleep as very poor in both stages of the research. Furthermore, rating sleep as very poor in 2022 resulted in a drop in points on the Resilience at Work Scale, compared to those who reported optimal sleep quality. A study conducted with the Brazilian population found that approximately 80% of participants had poor sleep quality, and 60% of the sample had low resilience^(21)^. Resilience strategies at work can also help in solving problems and managing daily stressors, which can consequently promote better sleep.

Workers who reported a significant impact of the pandemic on their mental health showed lower mean work resilience scores during and after the pandemic. Work resilience is fundamental in coping with unexpected situations, requiring a balance between physical and mental health, time for self-care, and analysis of adversities with a focus on positive solutions, supported by support networks. An international study^(22)^ with nurses from four countries indicated that organizational support and participation in the development of resilient strategies fostered higher levels of resilience. Conversely, nursing professionals working during the pandemic showed low levels of resilience and higher depression scores, with depression being directly impacted by levels of resilience at work^(15)^.

The workplace is also a fundamental component for maintaining mental health. Professionals who were reassigned from their sector after the pandemic demonstrated lower mean levels of resilience at work. A study on resilience for the Brazilian Unified Health System revealed that health institutions should have a contingency plan for staffing in crisis situations, including increasing or reallocating professionals^(23)^. However, it is important that this strategy be implemented with organizational support to maintain a healthy workforce.

During the pandemic, difficult decisions had to be made regarding sudden changes in care flows and the maintenance of care in response to the increased number of patients, primarily falling to institutional managers. In 2020, professionals holding leadership positions scored 13.53 points lower on the Resilience at Work Scale than those without management positions. Research conducted with nurses in leadership roles showed that resilience at work was essential to cope with the high levels of stress and challenges encountered in providing care and managing teams, inherent to leadership positions^(24)^.

Furthermore, among the work changes resulting from COVID-19, one of the established workflows was the creation of isolation units for patients who tested positive for the disease. In this study, professionals who worked in specific sectors for COVID-19 victims in 2022 scored, on average, 6.90 points higher on the Resilience at Work Scale compared to those who did not work in these sectors. This may be one of the consequences of the coping strategies developed during the pandemic. The study showed that professionals felt the impact of the pandemic on their physical and mental health similarly, both in units exclusively dedicated to treating COVID-19 cases and in other areas. However, team composition presented particularities, as well as distinct perceptions regarding occupational risks and work demands^(25)^.

Regarding worker illness after the pandemic, those who were absent due to suspected COVID-19 or other health reasons obtained lower resilience scores at work. A multicenter study^(22)^ conducted during the pandemic period revealed that the fear of becoming infected and testing positive for COVID-19 were inversely associated with resilience.

### Study limitations

A limitation of this study is the healthy worker effect bias, meaning that workers who remained active in the workforce cannot be measured, as it was not possible to assess the data of those who were absent due to health reasons during and after the pandemic. Future intervention studies are suggested to improve nursing professionals’ physical and mental health.

### Contributions to nursing, health, or public policy

Understanding the factors related to resilience in the workplace can help workers in their professional practice, because when they are healthy and have high levels of resilience, they are better able to cope with the adversities inherent in the profession, as well as having a lower chance of making mistakes and having accidents, resulting in greater safety for patients.

## CONCLUSIONS

During the pandemic, women and professionals in leadership positions showed lower levels of resilience at work. After the pandemic, this pattern was repeated among younger individuals, those with mental health diagnoses, those with poor sleep patterns, and those not working in COVID-19-specific sectors. These findings, when related to the theoretical framework of resilience, show that such vulnerabilities do not stem solely from individual characteristics, but primarily reflect organizational conditions and the demands imposed by the hospital work context.

Given this, the need for more structured institutional actions becomes evident, including continuous psychosocial support, initiatives aimed at promoting sleep and mental health, and strengthening support between managers and teams. Furthermore, strategies that improve working conditions and expand spaces for participation and cooperation can foster the development of resilience and reduce the risk of illness. Such measures are particularly relevant for groups identified as most susceptible, reinforcing the importance of public and institutional policies that consider, in an integrated manner, individual, organizational, and contextual factors.

## Data Availability

The research data are available only upon request.
