# Supplementary material for: Factors related to resilience in nursing workers during and after the pandemic: a prospective cohort
Source: Rev Bras Enferm. 2026 Sep 21;79:e20250396. doi: 10.1590/0034-7167-2025-0396 (PMC13592787; doi:10.1590/0034-7167-2025-0396)
Supplement: Translation

# Translation: Fatores relacionados à resiliência em trabalhadores de enfermagem durante e após a pandemia: coorte prospectiva

**DOI:** 10.1590/0034-7167-2025-0396pt

**Authors:** Luciana Olino, Miguel Lucas Silva da Paixão, Patrícia Bitencourt Toscani Greco, Tânia Solange Bosi de Souza Magnago, Daiane Dal Pai, Juliana Petri Tavares

**Affiliations:** I Universidade Federal do Rio Grande do Sul. Porto Alegre, Rio Grande do Sul, Brasil; II Universidade Federal do Rio Grande. Rio Grande, Rio Grande do Sul, Brasil; III Universidade Federal de Santa Maria. Santa Maria, Rio Grande do Sul, Brasil

**Keywords:** Resiliência, Enfermagem, COVID-19, Estudos de Coortes, Saúde Ocupacional.

## Abstract

**Objetivo:**

analisar os fatores que influenciam a resiliência no trabalho em profissionais de enfermagem que atuaram na área hospitalar durante e após a pandemia de COVID-19.

**Métodos:**

coorte prospectiva multicêntrica com 163 trabalhadores de enfermagem durante a após a pandemia. Coletaram-se variáveis sociodemográficas, laborais, de saúde e resiliência pela *Resilience at Work Scale* Brasil-25. Realizaram-se análise bivariada e modelo de regressão linear.

**Resultados:**

as médias de resiliência foram semelhantes em 2020 (73,3±11,1) e 2022 (72,9±11,2). Em relação ao escore, cada ano de idade aumentado acrescenta 0,27 pontos na Escala de Resiliência no Trabalho; ser do sexo feminino diminui 10,06 pontos; apresentar doença psíquica diminui 4,67 pontos; avaliar o sono como péssimo decresce 6,1 pontos; ocupar cargo de chefia diminui 13,53 pontos; atuar em setores específicos para vítimas da COVID-19 aumentou 6,90 pontos.

**Conclusão:**

embora semelhantes nos dois períodos, diferentes fatores influenciaram os escores de resiliência no trabalho.

## INTRODUÇÃO

O cotidiano dos profissionais da equipe de enfermagem é marcado por uma alta sobrecarga de trabalho, decorrente do frequente subdimensionamento das escalas, da superlotação dos serviços e da necessidade de estabelecer vínculos terapêuticos que garantam cuidado integral e seguro aos pacientes. Esse contexto incide diretamente sobre a saúde física e psíquica dos trabalhadores, aumentando a vulnerabilidade ao desenvolvimento de transtornos musculoesqueléticos e psicológicos^(1)^.

Na pandemia de COVID-19, o sistema de saúde entrou em colapso devido ao aumento no número e à complexidade dos casos. Essa situação resultou em uma sobrecarga de trabalho para os profissionais de saúde, especialmente para os trabalhadores da enfermagem, que constituem a maior parte dos recursos humanos nos serviços de saúde. Esse cenário acarretou cansaço, estresse e adoecimento psicológico entre esses profissionais^(2-5)^.

Nesse contexto, enfermeiros e técnicos necessitaram encontrar estratégias de adaptação para continuar exercendo a profissão. Nesse sentido, destaca-se a resiliência, compreendida como a capacidade de superar adversidades e eventos desagradáveis, bem como a habilidade de se adaptar com sucesso às mudanças e incertezas. Modelos clássicos de resiliência no trabalho enfatizam fatores individuais, como *coping* e autorregulação emocional, enquanto abordagens contemporâneas ampliam o conceito ao incluir dimensões organizacionais e contextuais, como suporte institucional, condições de trabalho, autonomia profissional e qualidade das relações interpessoais. Além disso, a resiliência contribui para a redução da exaustão emocional, para o aprimoramento do comprometimento e para a capacidade de lidar com as adversidades inerentes à profissão^(2,3,6-9)^.

Além do contexto pandêmico, os trabalhadores de enfermagem já enfrentavam desafios para desenvolver a resiliência no trabalho. Apesar dos cenários desafiadores enfrentados por esses trabalhadores, ainda são escassos na literatura os estudos de coorte que avaliem a resiliência no trabalho desses trabalhadores durante a pandemia e após pandemia. Estudo brasileiro^(10)^ destaca que, antes da pandemia, 15,2% da equipe de enfermagem apresentava baixos níveis de resiliência. No entanto, não há consenso na literatura que permita determinar como esses níveis se comportaram no período pós-pandêmico, evidenciando uma lacuna importante no conhecimento científico.

Diante desse contexto, torna-se necessário aprofundar o conhecimento sobre a importância da resiliência no ambiente de trabalho, que foi caracterizado por sobrecarga laboral e impactos na saúde física e psíquica. Além disso, embora os impactos da pandemia de COVID-19 sobre a saúde mental dos trabalhadores estejam amplamente documentados, persistem lacunas referentes aos fatores associados à resiliência e sua evolução ao longo do período pós-pandêmico, justificando a realização do presente estudo.

## OBJETIVOS

Analisar os fatores que influenciaram a resiliência no trabalho em trabalhadores de enfermagem que atuaram na área hospitalar durante e após a pandemia de COVID-19.

## MÉTODOS

### Aspectos éticos

Esta pesquisa foi aprovada pelo Comitê de Ética em Pesquisa e está de acordo com a Resolução n^o^ 466/12 do Conselho Nacional de Saúde, com a Resolução nº 510/16 sobre a utilização de dados obtidos diretamente com os participantes, e com as diretrizes da Lei Geral de Proteção de Dados, pois a privacidade dos participantes foi mantida. O Termo de Consentimento Livre e Esclarecido foi disponibilizado aos participantes via e-mail antes do preenchimento dos formulários.

### Desenho, período e local do estudo

Trata-se de estudo de coorte, multicêntrico, analítico e de abordagem quantitativa norteado pela ferramenta *STrengthening the Reporting of OBservational studies in Epidemiology* ^(11)^. Os participantes foram convidados a participar do estudo durante o pico pandêmico (fase 1), quando houve aumento da demanda por atendimentos hospitalares de pacientes com a doença, de agosto a outubro de 2020. Nessa etapa, participaram 817 profissionais. No segundo momento (fase 2), que ocorreu de agosto a dezembro de 2022, com estabilização da curva pandêmica caracterizada pelo decréscimo de novos casos, hospitalizações e óbitos pelo vírus, além da imunização populacional que torna controlada a pandemia, 382 trabalhadores de enfermagem preencheram o questionário.

Fizeram parte do estudo quatro instituições hospitalares do estado do Rio Grande do Sul, Brasil, que tiveram seus fluxos adaptados para o atendimento a pacientes acometidos pela COVID-19. Essas instituições incluem dois hospitais gerais e de ensino em Porto Alegre (HA, 784 leitos; HC, 850 leitos), um hospital público especializado em trauma agudo também localizado em Porto Alegre (HB, 237 leitos) e um hospital geral e de ensino situado em Santa Maria (HD, 403 leitos).

### População, amostra, critérios de inclusão e exclusão

A população do estudo foi constituída de 6.899 trabalhadores de enfermagem. Foram incluídos no estudo os trabalhadores de enfermagem (enfermeiros, técnicos e auxiliares de enfermagem) atuantes na assistência hospitalar durante a pandemia e que responderam ao formulário nos dois períodos de coleta (durante e após a pandemia). Foram excluídos aqueles profissionais que se afastaram por mais de 30 dias durante o período de coleta.

Foi calculado um tamanho amostral de 150 participantes para cada ano, a fim de testar se há diferença nos níveis de resiliência no trabalho entre a fase do pico pandêmico (2020) e o período posterior à pandemia (2022). O cálculo considerou um poder de 80%, um nível de significância de 5%, e percentuais de 32,2% (pré-pandêmico) e 48,5% (durante a pandemia), respectivamente, referidos na literatura^(12,13)^. A amostra do estudo de coorte foi composta de 163 trabalhadores da equipe de enfermagem, ou seja, indivíduos que participaram em ambas as fases do estudo.

### Protocolo do estudo

O instrumento de coleta de dados foi constituído no *Google Forms^®^*, abordando variáveis sociodemográficas como sexo, idade (data de nascimento), cor ou raça, número de filhos e situação conjugal. Outras variáveis referem-se aos hábitos de vida e saúde, como tabagismo, aumento do consumo de álcool, prática de atividade física, qualidade do sono, uso de medicações, ser portador de alguma doença e autoavaliação sobre o impacto da pandemia na saúde física e mental. Enviou-se o formulário via e-mail aos profissionais, sendo que o contato de e-mail do trabalhador foi acessado via autorização institucional. Além disso, houve divulgação interna nos setores dos hospitais participantes.

Foram coletados dados laborais como tempo de trabalho na profissão, cargo que ocupa, tipo de vínculo trabalhista, atuação em outra instituição, se possui cargo de chefia, turno de trabalho, setor de atuação, se trabalha na unidade específica para vítimas da COVID-19, se foi realocado durante a pandemia, se atendeu paciente com COVID-19, se os níveis de exigência do trabalho foram aumentados, nível de medo sentido diante da exposição ao risco de contaminação e se o funcionário necessitou de afastamento do trabalho por motivos de saúde e por suspeita ou confirmação da COVID-19.

A *Resilience at Work Scale* (RAW Scale) Brasil-25, validada no Brasil^(7)^, foi utilizada para avaliar a resiliência no trabalho. É uma escala Likert de sete pontos, com opções de 0 (Discordo totalmente) a 6 (Concordo totalmente). Avalia a resiliência no trabalho por intermédio das médias das respostas entre os sete domínios: vivendo autenticamente; encontrando vocação; mantendo equilíbrio; administrando o estresse; interagindo cooperativamente; mantendo-se saudável; e construindo redes^(6)^. A consistência interna da escala e de seus domínios foi avaliada pelo coeficiente de Cronbach, apresentando fidedignidade de 0,83. A confiabilidade composta variou de 0,56 a 0,85 entre os domínios^(7)^. Utilizaram-se os pontos de corte definidos pela autora da RAW *Scale* Brasil-25, cuja permissão para utilização foi concedida após contato com a mesma. Uma vez que tais informações são protegidas por direitos autorais, as mesmas não podem ser reproduzidas integralmente neste documento.

### Análises estáticas

Os dados de ambas as coletas foram captados do *Google Forms^®^* e enviadas para realização das respectivas análises no programa *Statistical Package for the Social Sciences* ^®^, versão 18. Utilizou-se o teste de Shapiro-Wilk para testar a normalidade. As variáveis categóricas foram apresentadas em frequência absoluta e relativa, e as variáveis contínuas, em tendência central e dispersão. Para comparar variáveis contínuas simétricas entre dois grupos independentes, empregou-se o teste t de *Student*, e para grupos dependentes, o teste t pareado. As variáveis paramétricas com três grupos ou mais foram submetidas à análise de variância.

Na análise múltipla, utilizaram-se a regressão linear, com variância robusta e expressa na razão de prevalência, e seus respectivos Intervalos de Confiança de 95% (IC95%). O critério de inserção das variáveis no modelo de regressão múltiplo final foi ter valor de p<0,20 tanto para a exposição (fatores associados) quanto para o desfecho (resiliência no trabalho). Foram consideradas como diferenças estatisticamente significativas os dados com IC95% ou com p bicaudal menor que 0,05.

## RESULTADOS

Participaram do estudo 163 trabalhadores de enfermagem, predominando o sexo feminino (n= 139; 85%) e a cor/raça branca (n=130; 79,8%) em ambas as etapas. Apresentaram mediana de idade de 43 (39-49) anos em 2020 e 44 (39-48) anos em 2022. Em 2020, 117 (71,8%) profissionais possuíam filhos, aumentando para 120 (73,6%) em 2022. Antes da pandemia, 126 (77,3%) eram casados, e após a pandemia, esse número reduziu para 124 (76,1%).

Quanto às variáveis laborais, 57,7% (n=94) possuíam cargo de enfermeiro, e 42,3% (n=69), de técnico e auxiliar de enfermagem. Quanto ao setor, a maioria atuava em unidades de internação adulto, sendo 46 (28,2%) em 2020 e 47 (28,8%) em 2022. O menor percentual de trabalhadores (n=56; 34,4%) não foi realocado de seu setor durante a pandemia em 2020, e 16 (9,81%), em 2022. Em 2020, a maioria trabalhava na instituição HC (n=85; 52,2%) no turno diurno (n=120; 73,6%), em regime de contratação conforme a Consolidação das Leis do Trabalho (n=139; 85,3%) e sem atividade em outra instituição (n=147; 90,2%). Em 2022, também prevaleceu a atuação na instituição HC (n=86; 52,8%) no turno diurno (n=109; 66,9%), em regime de contratação de acordo com a Consolidação das Leis do Trabalho (n=138; 84,7%) e sem atividade em outra instituição (n=150; 92%).

No que tange à resiliência no trabalho, em 2020, a média dos trabalhadores foi de 73,3 ±11,1; desses, 125 (76,7%) apresentaram resiliência no trabalho alta, e 38 (23,3%) manifestaram baixa resiliência no trabalho. Em 2022, o valor médio foi de 72,9 (±11,2). Desses, 122 (74,8%) trabalhadores se demonstraram com resiliência alta no trabalho, e 41 (25,2%), com resiliência baixa. A Tabela 1 apresenta a distribuição da resiliência no trabalho (RAW *Scale* Brasil-25) dos trabalhadores de enfermagem (n=163) atuantes no período durante e após a pandemia, segundo características sociodemográficas, hábitos de vida e saúde.

**Tabela 1:** Distribuição das médias de resiliência (RAW *Scale* Brasil-25) dos trabalhadores de enfermagem (N=163) atuantes no período durante e após a pandemia segundo características sociodemográficas, hábitos de vida e saúde, Porto Alegre, Rio Grande do Sul, Brasil, 2020 e 2022

Variáveis sociodemográficas, hábitos de vida e saúde		Ano	
		2020	2022
Idade (em anos)^*^		R=0,19	R=0,20
		p=**0,017**	p=**0,011**
Sexo	Feminino	72,33 ±11,29	71,88 ±12,23
	Masculino	78,28 ±9,28	73,19 ±11,96
		**p=0,008**	p=0,624
Cor/raça	Branca	73,21 ±10,63	72,29 ±10,69
	Não branca	73,19 ±13,36	71,21 ±16,97
		p=0,993	p=0,650
Presença de filhos	Sim	73,17 ±10,58	72,06 ±12,43
	Não	73,30 ±12,73	72,11 ±11,55
		p=0,949	p=0,982
Situação conjugal	Solteiro	73,28 ±10,44	72,60 ±12,23
	Casado	73,19 ±11,44	71,91 ±12,19
		p=0,963	p=0,760
Tabagismo	Sim	77,27 ±8,25	73,23 ±9,39
	Não	72,94 ±11,33	71,97 ±12,40
		p=0,145	p=0,659
Aumento do consumo de álcool nos últimos seis meses	Sim	73,18 ±10,58	69,61 ±13,17
	Não	73,95 ±11,36	72,61 ±11,92
		p=0,149	p=0,266
Prática de atividade física nos últimos seis meses	Sim	77,96 ±8,30	73,55 ±12,14
	Não	71,33 ±11,65	70,2 ±12,03
		p=**<0,001**	p=0,081
Medicação iniciada nos últimos seis meses	Sim	71,33 ±12,20	67,17 ±14,16
	Não	73,9 ±10,77	73,67 ±11,04
		p=0,223	p=**0,003**
Presença de alguma doença	Sim	72,23 ±12,14	71,42 ±12,02
	Não	74,31 ±9,99	72,86 ±12,37
		p=0,229	p=0,452
Doenças cardiovasculares	Sim	74,41 ±11,17	73,56 ±11,91
	Não	72,95 ±11,22	71,67 ±12,25
		p=0,525	p=0,411
Doenças endócrinas	Sim	70,11 ±12,43	67,18 ±16,92
	Não	73,45±11,10	72,5 ±11,64
		p=0,384	p=0,287
Doenças psíquicas	Sim	65,68 ±11,11	64,92 ±11,77
	Não	74,44 ±10,75	73,62 ±11,73
		p=**0,001**	p=**0,001**
Doenças musculoesqueléticas	Sim	71,59 ±12,24	68,41 ±11,76
	Não	73,54 ±10,98	73,04 ±12,13
		p=0,441	p=**0,048**
Doenças digestivas	Sim	70,77 ±12,05	68,49 ±11,6
	Não	73,54 ±11,07	72,55 ±11,62
		p=0,339	p=0,287
Doenças respiratórias	Sim	73,21 ±11,45	70,86 ±9,83
	Não	73,21 ±11,20	72,21 ±12,43
		p=0,998	p=0,608
Qualidade do sono nos últimos seis meses	Ótima	75,10 ±10,41	73,7 ±11,30
	Péssima	68,22 ±11,74	65,51 ±13,18
		p=**0,001**	p=**0,001**
Impacto da pandemia de COVID-19 na saúde física	Intenso	72,04 ±11,11	70,63 ±12,51
	Nenhum	74,58 ±11,21	73,24 ±11,82
		p=0,150	p=0,176
Impacto da pandemia de COVID-19 na saúde mental	Intenso	71,33 ±11,22	70,34 ±11,91
	Nenhum	78,60 ±9,3	74,26 ±12,22
		p=**<0,001**	p=**0,042**

* *Valores de correlação de Pearson (R); p - valor de p.*

Em 2020, associaram-se a menores médias de resiliência (p<0,05): idade mais jovem (R=0,19); sexo feminino (72,33 ± 11,29) em relação ao masculino (78,28 ± 9,28); sedentarismo (71,33 ± 11,65); presença de doença psíquica (65,68 ± 11,11); qualidade do sono péssima (68,22 ± 11,74); e percepção de impacto intenso da pandemia na saúde mental (71,33 ± 11,22).

Em 2022, observaram-se menores escores de resiliência entre trabalhadores mais jovens (R=0,20), que iniciaram medicação nos últimos seis meses (67,17 ± 14,16), portadores de doenças psíquicas (64,92 ± 11,77) ou musculoesqueléticas (68,41 ± 11,76), com sono avaliado como péssimo (65,51 ± 13,18) e entre aqueles que relataram impacto intenso da pandemia na saúde mental (70,34 ± 11,9). As características laborais e os índices de resiliência dos trabalhadores estão detalhados na Tabela 2.

**Tabela 2:** Distribuição das médias de resiliência no trabalho (RAW *Scale* Brasil-25) dos trabalhadores de enfermagem (N=163) atuantes no período durante e após a pandemia segundo características laborais, Porto Alegre, Rio Grande do Sul, Brasil, 2020 e 2022

Características laborais		Ano	
		2020	2022
Tempo em que atua na profissão (anos)^*^		R=0,07 p=0,392	R=0,05 p=0,496
Cargo	Enfermeiro	72,57 ±11,29	72,02 ±10,91
	Técnico de enfermagem	73,77 ±10,38	72,3 ±13,73
	Auxiliar de enfermagem	77,22 ±18,03	70,56 ±15,52
		p=0,612	p=0,944
Tipo de vínculo trabalhista	Celetista	73,59 ±11,13	72,32 ±11,81
	Estatutário	70,64 ±11,69	70,72 ±14,15
		p=0,449	p=0,547
Atua em outra instituição	Sim	73,92 ±12,77	73,84 ±12,21
	Não	73,13 ±11,05	71,92 ±12,19
		p=0,815	p=0,586
Cargo de chefia	Sim	77,16 ±9,45	72,70 ±9,6
	Não	72,81 ±11,31	72 ±12,47
		p=0,113	p=0,778
Turno de trabalho	Diurno	72,96 ±11,83	71,42 ±12,26
	Noturno	75,21 ±8,7	74,41 ±12,26
	Sem turno fixo	68,96 ±10,18	67,5 ±8,56
		p=0,296	p=0,210
Setor de trabalho	Emergência	76,37 ±8,71	70,63 ±10,4
	Terapia intensiva	72,21 ±12,74	74,43 ±9,53
	Internação	72,01 ±9,95	73,79 ±12,9
	Centro cirúrgico	77 ±8,86	73,46 ±10,31
	Pediatria e neonatologia	70,17 ±14,09	69,28 ±12,39
	Demais setores	73,20 ±12,20	69,99 ±13,7
		p=0,444	p=0,532
Realocado de seu setor nos últimos seis meses	Sim	73,64 ±11,54	65,79 ±12,41
	Não	72,97 ±11,05	72,76 ±11,98
		p=0,724	p=**0,046**
Setor de trabalho específico para vítimas da COVID-19	Sim	72,43 ±10,66	77,60 ±11,84
	Não	73,48 ±11,41	71,51 ±12,10
		p=0,591	p=0,075
Atendeu paciente com suspeita ou diagnóstico da COVID-19 nos últimos seis meses	Sim	73,14 ±11,01	71,64 ±12,46
	Não	73,68 ±11,82	74,07 ±10,70
		p=0,862	p=0,288
Medo sentido diante da exposição ao risco de contaminação nos últimos seis meses	Intenso	71,88 ±11,19	71,75 ±11,83
	Pouco	75,06 ±11,01	72,17 ±12,31
		p=0,075	p=0,851
Aumento dos níveis de exigência do seu trabalho nos últimos seis meses	Intenso	73,23 ±10,41	72,08 ±11,34
	Pouco	72,9 ±13,96	72,06 ±13,57
		p=0,881	p=0,989
Afastamento do trabalho por motivo de saúde nos últimos seis meses	Sim	71,79 ±11,93	68,93 ±12,54
	Não	74,33 ±10,51	75,03 ±11,09
		p=0,157	p=**0,001**
Afastamento do trabalho por suspeita da COVID-19 nos últimos seis meses	Sim	73,29 ±11,53	68,56 ±11,71
	Não	73,14 ±10,98	74,12 ±12,01
		p=0,934	p=**0,004**
Afastamento do trabalho por diagnóstico da COVID-19 nos últimos seis meses	Sim	73,4 ±12,41	70,26 ±11,05
	Não	73,18 ±11,05	72,79 ±12,55
		p=0,940	p=0,210

* *Valores de correlação de Pearson (R); p - valor de p.*

Ter sido realocado de seu setor em 2022, ser afastado do seu trabalho por motivo de saúde e suspeita da COVID-19 se relacionaram a menores médias de resiliência (p<0,05). Na Tabela 3, estão apresentados os níveis categorizados de resiliência no trabalho.

**Tabela 3:** Trabalhadores de enfermagem (N=163) atuantes no período durante e após a pandemia segundo os níveis alto e baixo de resiliência no trabalho entre os domínios da escala, Porto Alegre, Rio Grande do Sul, Brasil, 2020 e 2022

Domínio		Ano	
		2020	2022
Vivendo autenticamente	Alta	95,1 (155)	93,3 (152)
	Baixa	4,9 (8)	6,7 (11)
Encontrando vocação	Alta	86,5 (141)	85,3 (139)
	Baixa	13,5 (22)	14,7 (24)
Mantendo equilíbrio	Alta	20,9 (34)	25,2 (41)
	Baixa	79,1 (129)	74,8 (122)
Administrando o estresse	Alta	60,7 (99)	60,1 (98)
	Baixa	39,3 (64)	39,9 (65)
Interagindo cooperativamente	Alta	73,6 (120)	76,1 (124)
	Baixa	26,4 (43)	23,9 (39)
Mantendo-se saudável	Alta	65,6 (107)	63,2 (103)
	Baixa	34,4 (56)	36,8 (60)
Construindo redes	Alta	80,4 (131)	76,7 (125)
	Baixa	19,6 (32)	23,3 (38)

No que se refere à resiliência, em 2020, a média dos trabalhadores foi de 72,1 (±12,2). Em 2022, o valor médio foi de 72,9 (±11,2). Na Tabela 3, estão apresentados os trabalhadores de enfermagem atuantes no período durante e após a pandemia segundo os domínios da resiliência. O modelo de regressão linear para as variáveis selecionadas à resiliência está apresentado na Tabela 4.

**Tabela 4:** Modelo de regressão linear para variáveis relacionadas à resiliência no trabalho (N=163), Porto Alegre, Rio Grande do Sul, Brasil 2022

Variáveis		2020	2022
		Razão de prevalência (IC95%)	Razão de prevalência (95%IC)
Idade		0,14 (-0,25 a 0,53)	0,267 (0,06 a 0,48)
		p=0,471	p=**0,013**
Sexo	Feminino	-10,06 (-18,85 a 1,27)	N/A
	Masculino	1	N/A
		p=**0,025**	N/A
Tabagismo	Sim	5,02 (-5,02 a 15,42)	N/A
	Não	1	N/A
		p=0,319	N/A
Aumento do consumo de álcool nos últimos seis meses	Sim	3,83 (-1,51 a 9,17)	N/A
	Não	1	N/A
		p=0,159	N/A
Prática de atividade física nos últimos seis meses	Sim	1	1
	Não	-7,35 (-0,35 - 15,1)	-1,87 (-1,53 a -5,27)
		p=0,062	p=0,282
Medicação iniciada nos últimos seis meses	Sim	N/A	-2,66 (-6,62 a 1,3)
	Não	N/A	1
		N/A	p=0,188
Doenças psíquicas	Sim	-2,56 (-9 a 3,86)	-4,67 (-9,33 a -0,23)
	Não	1	1
		p=0,435	p=**0,049**
Doenças musculoesqueléticas	Sim	N/A	-1,62 (-5,8 a 2,56)
	Não	N/A	1
		N/A	0,447
Qualidade do sono nos últimos seis meses	Péssima	-1,82 (-7,51 a 3,86)	-6,08 (-10,88 a -1,27)
	Ótima	1	1
		p=0,530	p=**0,013**
Impacto da pandemia de COVID-19 na sua saúde física	Intenso	1,61 (-3,97 a 7,2)	0,8 (-2,89 a 4,49)
	Nenhum	1	1
		p=0,571	p=0,671
Impacto da pandemia de COVID-19 na sua saúde mental	Intenso	2,19 (-8,9 - 13,29)	-1,45 (-5,21 a 2,31)
	Nenhum	1	1
		p=0,700	0,451
Cargo de chefia	Sim	-13,53 (-26,08 a -0,99)	N/A
	Não	1	N/A
		p=0,034	N/A
Realocado de seu setor nos últimos seis meses	Sim	N/A	-3,62 (-9,2 a 1,95)
	Não	N/A	1
		N/A	p=0,203
Setor de trabalho específico para vítimas da COVID-19	Sim	N/A	6,90 (1,22 a 12,58)
	Não	N/A	1
		N/A	p=0,017
Medo sentido diante da exposição ao risco de contaminação nos últimos seis meses	Intenso	-3,75 (-10,69 a 3,20)	N/A
	Pouco	1	N/A
		p=0,290	N/A
Afastamento do trabalho por motivo de saúde nos últimos seis meses	Sim	1,10 (-4,37 a 6,56)	-3,08 (-6,98 a 0,82)
	Não	1	1
		p=0,694	p=0,122
Afastamento do trabalho por suspeita da COVID-19 nos últimos seis meses	Sim	Não se aplica	-1,32 (-5,36 a 2,73)
	Não	Não se aplica	1
		Não se aplica	p=0,523

*IC - Intervalo de Confiança; p - valor de p; N/A - não se aplica.*

Na análise de regressão linear das variáveis relacionadas à resiliência, em 2020, ter sexo feminino diminuiu, em média, 10,06 pontos na Escala de Resiliência no Trabalho em comparação ao sexo masculino (p=0,025). Ocupar cargo de chefia pontuou 13,53 pontos a menos na Escala de Resiliência no Trabalho em comparação com aqueles que não possuíam cargo de gestão (p=0,034). Em 2022, a cada ano aumentado de idade, o indivíduo obteve, aproximadamente, 0,27 pontos a mais na Escala de Resiliência no Trabalho (p=0,013); possuir doença psíquica diminuiu 4,67 pontos na Escala de Resiliência no Trabalho em comparação com quem não possuía (p=0,049); quem avaliou a qualidade do sono como péssima teve um decréscimo de, aproximadamente, 6,1 pontos na Escala de Resiliência no Trabalho em comparação com quem relatou qualidade do sono como ótima (p=0,013); atuar em setores específicos para vítimas da COVID-19 obteve, em média, 6,90 pontos a mais na Escala de Resiliência no Trabalho em comparação com quem não trabalhava (p=0,017).

## DISCUSSÃO

A média global de resiliência no trabalho foi discretamente maior durante a pandemia do que após seu término. Provavelmente, isso ocorreu porque os trabalhadores necessitavam buscar veementemente forças e subsídios para equilibrar os danos que estavam vivenciando no ano de 2020, o que em 2022 já havia uma estabilidade e, consequentemente, menor necessidade de adaptação. Estudo realizado na Bélgica, fora do contexto pandêmico, detectou menor resiliência no trabalho, além de carência de atividades para reduzir o estresse, que esteve diretamente associado à tentativa de suicídio^(14)^. Estudo realizado no contexto pandêmico com profissionais de enfermagem de todas as regiões do Brasil demonstrou que os participantes tiveram, em geral, maiores pontuações médias para depressão atrelados a baixos níveis de resiliência e autoeficácia^(15)^.

A prevalência de níveis altos e baixos de resiliência no trabalho se manteve semelhante entre os domínios da escala durante os períodos pandêmico e pós-pandêmico. A dimensão com maior pontuação em ambos os anos foi “Vivendo autenticamente”, relacionada ao autoconhecimento, regulação emocional e alinhamento de valores pessoais e institucionais, o que favorece o manejo do humor e da pressão^(6)^. Já o domínio com menor escore foi “Mantendo equilíbrio”, que envolve lidar com adversidades e manter positividade diante de situações negativas - aspecto possivelmente prejudicado pelas dificuldades enfrentadas durante e após a pandemia. Apoio organizacional e planejamento são fundamentais para fortalecer essa dimensão^(6)^.

Evidenciou-se que, após a pandemia, as médias de resiliência no trabalho foram maiores nos indivíduos mais velhos. Tais dados se assemelham ao estudo realizado em Santa Catarina, Brasil, durante a pandemia, que demonstrou haver diferença estatisticamente significativa para maior pontuação de resiliência entre as pessoas com mais de 35 anos, em comparação com as de outras faixas etárias menores^(16)^. É possível pensar que quanto maior a idade e a experiência profissional, melhor é a capacidade analítica da situação para superar as adversidades e, consequentemente, maior seria o arcabouço de estratégias para o enfrentamento das adversidades, devido às experiências previamente vivenciadas.

Em 2020, os piores níveis de resiliência no trabalho foram observados no sexo feminino. Estudo sobre resiliência descreveu resultados semelhantes, no qual profissionais do sexo masculino tiveram escore de resiliência estatisticamente maior do que trabalhadoras do sexo feminino. A categoria enfermeiro teve escore de resiliência estatisticamente maior do que a categoria técnico de enfermagem^(15)^.

Os profissionais de enfermagem que não praticavam atividade física durante a pandemia e também aqueles que tinham doenças musculoesqueléticas após a pandemia apresentaram menores médias de resiliência no trabalho. Estudo sobre resiliência, desenvolvido com a equipe de enfermagem de um hospital geral, constatou que 26,6% dos participantes apresentaram baixa resiliência, 40% apresentaram resiliência moderada, 33,4% apresentaram resiliência alta, e somente 33,3% dos respondentes praticavam atividade física regularmente. A pesquisa também evidenciou que a intensidade da dor musculoesquelética foi de moderada a alta nos participantes, em diferentes regiões anatômicas^(17)^.

Quanto ao início do uso de medicações, aqueles profissionais que iniciaram o uso após a pandemia apresentaram menores médias de resiliência no trabalho. Estudo brasileiro sobre resiliência descreveu que os participantes que nunca utilizaram medicações são os que possuem maior resiliência, com diferença estatisticamente significativa nas comparações das pontuações^(18)^.

O benefício da resiliência no trabalho como fator protetor de doenças psiquiátricas é conhecido através da literatura^(19)^. No presente estudo, as menores médias de resiliência estavam presentes nos trabalhadores que portavam doenças psíquicas, bem como ter doença psíquica, em 2022, diminuiu pontos na Escala de Resiliência no Trabalho. Os dados corroboram o estudo que evidenciou correlação estatisticamente significativa negativa entre a depressão e a resiliência no trabalho, mostrando que quanto maior a resiliência, menor a depressão^(20)^. Reforçou-se a importância de desenvolver estratégias resilientes como ferramenta de tratamento e prevenção do adoecimento psicológico.

Além de doenças psíquicas, a resiliência no trabalho diminuída pode afetar o sono. Neste estudo, os profissionais que apresentaram menores médias de resiliência no trabalho foram aqueles que avaliaram seu sono como péssimo em ambas as etapas da pesquisa. Além disso, avaliar o sono como péssimo em 2022 resultou em uma queda de pontos na Escala de Resiliência no Trabalho, em comparação com os que relataram qualidade de sono ótima. Estudo realizado com a população brasileira constatou que aproximadamente 80% dos participantes possuíam baixa qualidade de sono, e 60% da amostra possuíam baixa resiliência^(21)^. As estratégias de resiliência no trabalho também são capazes de auxiliar na solução de problemas e na administração dos fatores estressores do cotidiano, o que consequentemente pode favorecer um sono melhor.

Trabalhadores que relataram intenso impacto da pandemia na saúde mental apresentaram menores médias de resiliência no trabalho durante e após o período pandêmico. A resiliência laboral é fundamental no enfrentamento de situações inesperadas, exigindo equilíbrio entre saúde física e mental, tempo para autocuidado e análise das adversidades com foco em soluções positivas, apoiado por redes de suporte. Estudo internacional^(22)^ com enfermeiros de quatro países indicou que apoio organizacional e participação no desenvolvimento de estratégias resilientes favoreceram maiores níveis de resiliência. Por sua vez, profissionais de enfermagem atuantes na pandemia apresentaram baixos níveis de resiliência e maiores escores de depressão, sendo esta impactada diretamente pelos níveis de resiliência no trabalho^(15)^.

O setor de trabalho também se faz componente fundamental para a manutenção da saúde psíquica. Os profissionais que foram realocados de seu setor após a pandemia demonstraram menores médias de resiliência no trabalho. Estudo realizado sobre resiliência para o Sistema Único de Saúde divulgou que as instituições de saúde devem possuir plano de contingência para provimento de pessoal em situação de crise, com aumento ou realocação de profissionais^(23)^; porém, faz-se importante que essa estratégia seja aplicada com apoio organizacional para manutenção saudável da força de trabalho.

No momento da pandemia, decisões difíceis precisaram ser tomadas no que tange às mudanças repentinas nos fluxos de atendimento e manutenção da assistência ao acréscimo do número de doentes, ficando a cargo, principalmente, dos gestores institucionais. No ano de 2020, os profissionais que ocupavam cargo de chefia pontuaram 13,53 pontos a menos na Escala de Resiliência no Trabalho do que aqueles que não possuíam cargo de gestão. Pesquisa realizada com enfermeiros que possuíam cargo de liderança evidenciou que a resiliência no trabalho foi indispensável para enfrentar o alto nível de estresse e os desafios encontrados na assistência e na gestão das equipes, intrínsecos ao cargo de chefia^(24)^.

Ademais, entre as mudanças de trabalho provenientes da COVID-19, um dos fluxos estabelecidos foi a criação de unidades de isolamento para os pacientes positivos para a doença. Neste estudo, os profissionais que atuavam nos setores específicos para vítimas da COVID-19, em 2022, obtiveram, em média, 6,90 pontos a mais na Escala de Resiliência no Trabalho em comparação com quem não trabalhava. É possível que esta seja uma das consequências das estratégias de enfrentamento desenvolvidas durante a pandemia. Estudo evidenciou que os profissionais sentiram semelhantemente o impacto da pandemia na sua saúde física e psíquica, tanto nas unidades exclusivas para atendimento de casos da COVID-19 quanto nas demais áreas. Entretanto, a conformação das equipes apresentou particularidades, bem como percepções distintas em relação aos riscos ocupacionais e às exigências de trabalho^(25)^.

Referente ao adoecimento dos trabalhadores após a pandemia, aqueles que se afastaram por suspeita de COVID-19 ou por outros motivos de saúde obtiveram pontuações mais baixas de resiliência no trabalho. Estudo multicêntrico^(22)^ realizado durante o período da pandemia revelou que o medo de se infectar e ter teste positivo para COVID-19 estiveram inversamente associados à resiliência.

### Limitações do estudo

Como limitação do estudo, observa-se o viés de efeito do trabalhador sadio, ou seja, daqueles que permaneceram ativos na força de trabalho, não sendo possível mensurar os dados daqueles que se afastaram por motivos de saúde durante e após a pandemia. Sugere-se, no futuro, estudos de intervenção com vistas a melhorar a saúde física e mental dos profissionais de enfermagem.

### Contribuições para a área da enfermagem, saúde ou políticas públicas

A compreensão acerca dos fatores relacionados à presença de resiliência no trabalho pode auxiliar os trabalhadores em sua prática profissional, pois quando saudáveis e com níveis elevados de resiliência, apresentam melhor enfrentamento das adversidades inerentes à profissão, bem como menores chances de cometer erros e acidentes, refletindo em maior segurança aos pacientes.

## CONCLUSÕES

Durante a pandemia, mulheres e profissionais em cargos de chefia apresentaram menores níveis de resiliência no trabalho. Após a pandemia, esse padrão se repetiu entre os mais jovens, com diagnóstico psíquico, com sono avaliado como péssimo e que não atuavam em setores específicos para COVID-19. Esses achados, quando relacionados ao referencial teórico da resiliência, mostram que tais vulnerabilidades não decorrem apenas de características individuais, mas refletem, sobretudo, as condições organizacionais e as exigências impostas pelo contexto de trabalho hospitalar.

Diante disso, torna-se evidente a necessidade de ações institucionais mais estruturadas, que incluam apoio psicossocial contínuo, iniciativas voltadas à promoção do sono e da saúde mental, e o fortalecimento do suporte entre chefias e equipes. Além disso, estratégias que qualifiquem as condições de trabalho e ampliem espaços de participação e cooperação podem favorecer o desenvolvimento da resiliência e reduzir o risco de adoecimento. Tais medidas se mostram particularmente relevantes para os grupos identificados como mais suscetíveis, reforçando a importância de políticas públicas e institucionais que considerem, de maneira integrada, fatores individuais, organizacionais e contextuais.

## Data Availability

Os dados de pesquisa só estão disponíveis mediante solicitação.
